# Altered Gene Expression in the Schistosome-Transmitting Snail *Biomphalaria glabrata* following Exposure to Niclosamide, the Active Ingredient in the Widely Used Molluscicide Bayluscide

**DOI:** 10.1371/journal.pntd.0004131

**Published:** 2015-10-09

**Authors:** Si-Ming Zhang, Sarah K. Buddenborg, Coen M. Adema, John T. Sullivan, Eric S. Loker

**Affiliations:** 1 Center for Evolutionary and Theoretical Immunology, Department of Biology, The University of New Mexico, Albuquerque, New Mexico, United States of America; 2 Department of Biology, University of San Francisco, San Francisco, California, United States of America; 3 Parasite Division, Museum of Southwestern Biology, Department of Biology, The University of New Mexico, Albuquerque, New Mexico, United States of America; George Washington University, UNITED STATES

## Abstract

In view of the call by the World Health Organization (WHO) for elimination of schistosomiasis as a public health problem by 2025, use of molluscicides in snail control to supplement chemotherapy–based control efforts is likely to increase in the coming years. The mechanisms of action of niclosamide, the active ingredient in the most widely used molluscicides, remain largely unknown. A better understanding of its toxicology at the molecular level will both improve our knowledge of snail biology and may offer valuable insights into the development of better chemical control methods for snails. We used a recently developed *Biomphalaria glabrata* oligonucleotide microarray (31K features) to investigate the effect of sublethal exposure to niclosamide on the transcriptional responses of the snail *B*. *glabrata* relative to untreated snails. Most of the genes highly upregulated following exposure of snails to niclosamide are involved in biotransformation of xenobiotics, including genes encoding cytochrome P450s (CYP), glutathione S-transferases (GST), and drug transporters, notably multi-drug resistance protein (efflux transporter) and solute linked carrier (influx transporter). Niclosamide also induced stress responses. Specifically, six heat shock protein (HSP) genes from three super-families (HSP20, HSP40 and HSP70) were upregulated. Genes encoding ADP-ribosylation factor (ARF), cAMP response element-binding protein (CREB) and coatomer, all of which are involved in vesicle trafficking in the Golgi of mammalian cells, were also upregulated. Lastly, a hemoglobin gene was downregulated, suggesting niclosamide may affect oxygen transport. Our results show that snails mount substantial responses to sublethal concentrations of niclosamide, at least some of which appear to be protective. The topic of how niclosamide’s lethality at higher concentrations is determined requires further study. Given that niclosamide has also been used as an anthelmintic drug for decades and has been found to have activity against several types of cancer, our findings may be of relevance in understanding how both parasites and neoplastic cells respond to this compound.

## Author Summary

Schistosomes are snail-transmitted parasites that continue to infect over 230 million people worldwide and cause the disease schistosomiasis. Currently there is no effective vaccine against the disease. Control programs have relied primarily on use of chemotherapy with praziquantel to eliminate adult worms from infected people. An increasing body of evidence, however, suggests that praziquantel-based control programs are not likely to be sufficient to achieve sustainable transmission control. Snail control achieved by focal use of molluscicides, especially in combination with other methods like chemotherapy, sanitation and health education, offers considerable promise for reduction of disease transmission. Consequently, use of molluscicides in snail control is likely to increase in the coming years. We undertook a microarray study to assess transcriptional responses to niclosamide, the active ingredient in commonly-used molluscicides, in the schistosome-transmitting snail *Biomphalaria glabrata*. We show that niclosamide activates components in snails’ pathways known to be involved in biotransformation of xenobiotics and stress responses. We suggest that major alterations in vesicle trafficking and interference with oxygen transport also follow niclosamide exposure. The results contribute to our understanding of molecular impacts of niclosamide exposure on snails, and provide a basis for further studies to define the mode of action of niclosamide and other molluscicides in the future.

## Introduction

Schistosomiasis, caused by blood-dwelling digenetic trematodes of the genus *Schistosoma*, is one of the world’s major neglected tropical diseases. By conservative estimates, at least 230 million people worldwide are infected with *Schistosoma* spp. [1]. Among eukaryotic parasites, the global health impact of schistosomiasis is second only to malaria.

Schistosomes have an indirect life cycle, involving asexual reproduction in a snail intermediate host and sexual reproduction in a mammalian or avian definitive host. Use of praziquantel to kill adult worms has been a mainstay of schistosomiasis control for about forty years [2]. However, an increasing body of evidence suggests that praziquantel-based control programs are not likely to be sufficient to achieve sustainable transmission control [3–5].

Recently, King and Bertsch (2015) reviewed the application of molluscicides around the world and re-emphasized their importance in schistosomiasis control [6]. Snail control achieved by focal use of molluscicides, especially in combination with other methods like chemotherapy, sanitation and health education, offers considerable promise for reduction of disease transmission.

For the past half-century, snail control has relied primarily on a single compound, namely, niclosamide. Niclosamide was selected as a molluscicide in the 1950s after screening of well over 20,000 compounds for toxicity against the schistosome-transmitting snail *Biomphalaria glabrata* [7]. Currently, Bayluscide, containing niclosamide or its ethanolamine salt, is still being applied in many endemic areas, mostly in Africa and Asia [8–12].

A limited number of early studies based on physiological and biochemical assays suggested that niclosamide affects snail oxygen consumption and carbohydrate metabolism. High concentrations of niclosamide (above 0.15mg/L) reduce oxygen uptake whereas low concentrations increase oxygen uptake [13]. Niclosamide may also interfere with glucose metabolism [14,15]. Nevertheless, the underlying mechanism of niclosamide’s potent activity in killing snails remains unclear even though its molluscicidal properties were revealed over sixty years ago [7].

Given the concerns about the sustainability of chemotherapy-based control, potential emergence of resistance to praziquantel, and lack of an anti-schistosome vaccine in the near future, development of additional methods of snail control, including a new generation of highly specific, environmentally friendly molluscicides, is a high priority in light of WHO’s call for elimination of schistosomiasis where possible by 2025 [16]. As shown from a number of studies of the effects of pesticides on insect disease vectors [17–19], understanding the toxicology of niclosamide in snails would be helpful in developing effective new molluscicides, ultimately benefiting schistosomiasis control.

In addition to its potent molluscicidal activity, niclosamide has also been used as an anthelmintic drug for treatment of adult tapeworm infection for decades [20]. Interference with mitochondrial oxidative phosphorylation is believed to play a role in niclosamide’s anthelmintic effect [21–23]. However, no further validation or investigation of its effects on tapeworms has been undertaken, despite the availability of new molecular-based biotechnologies. Recent studies have demonstrated that niclosamide has activity against a variety of cancer cells including those of prostate, breast, ovarian, colon, lung, and head and neck cancers, in part by suppressing various intracellular signaling pathways, including Wnt/beta-catenin, NOTCH, mTORC1, and NF-κB [24, 25]. Niclosamide also has activity against rhinovirus and influenza viruses [26]. It is also noteworthy to mention that niclosamide has an excellent safety profile in humans [7, 27].

Documenting the impact of niclosamide by monitoring transcriptomic responses of target species like snails can help reveal both the nature of the protective responses mounted and yield clues to the underlying basis of its action. In this study, we employed an oligonucleotide microarray to investigate the transcriptomic response of the schistosome-transmitting snail *Biomphalaria glabrata* to a 24-hr exposure to water containing three different sublethal concentrations of niclosamide.

## Materials and Methods

### Snails

All snails used in this study were laboratory-reared *Biomphalaria glabrata* of the M-line strain [28]. Snails were fed lettuce *ad libitum* and maintained on a 12 h light: 12 h dark schedule in artificial spring water [29]. Snails of 8–11mm shell diameter were used.

### Treatment of snails with niclosamide

Niclosamide was purchased from Sigma and dissolved in dimethyl sulfoxide (DMSO) (Sigma). A volume of 2L of artificial spring water was added to each of four 3L plastic containers. Also, each container also received a total of 32–34 snails. Three niclosamide concentrations, 0.15 mg/L, 0.10 mg/L, and 0.05 mg/L, were tested. DMSO vehicle alone was added to the fourth, control container. The final DMSO concentration in each of the four containers was the same, at 1/1000 (v/v). Snails were exposed to niclosamide for 24 hours at 26–28°C with aeration. After the 24-hour exposure, twenty live snails from each container were collected and randomly divided into four replicate groups, each with five snails, for subsequent RNA extraction. For each of the three treatment groups and the control, the four replicate groups (five snails each) that were collected are considered to constitute valid biological replicates because each replicate is comprised of a distinct set of five snails. Furthermore, our design ensured that exposure conditions for a particular concentration were identical for each replicate.

We define the concentrations used as “sublethal” because the snails we sampled were alive and responsive after the 24 hour period of niclosamide exposure. It is possible that some of the snails selected for study would have died upon further observation, but had not done so at the time of sampling.

### Extraction, qualification and quantitation of RNA

For a particular treatment, whole bodies of five snails dissected from their shells were pooled as a single sample, and ground in liquid nitrogen. Two sequential RNA extraction methods were applied to each such sample, first using Trizol (Invitrogen) and then the PureLink RNA kit (Ambion), following manufacturers’ instructions. Quality and quantity of RNA were checked using an Agilent Bioanalyzer 1200 and NanoDrop spectrophotometer and quality was determined to be high (sharply focused rRNA bands and A260/A280 ratio: 1.9–2.1) for all samples.

### Description of microarray

The microarray used for this study contains 60-mer oligonucleotide probes that were designed using transcriptomic sequence data from *B*. *glabrata* from publically available databases in 2010. Sequences were computationally assembled to unique predicted transcripts using a method described [30] and used to design probe sequences and a microarray (duplicate probes were arranged in 8 X 60K layout) using the eArray facilities from Agilent. The details of probes, target sequences and microarray design (eArray AMADID 033677) are available at Gene Expression Omnibus (GEO) at Platform Access Number GPL20716 (www.ncbi.nih.gov/geo/). Compared to recent microarray designs (i.e., arrays with 1.1K oligonucleotide probes [31] and 5K cDNA probes [32]), this 31K microarray design provides the most comprehensive coverage of expressed sequences from *B*. *glabrata* to date.

### Microarray hybridization

To provide a positive hybridization signal to as many elements of the array as possible, universal reference RNA (URR) consisted of 80% RNA from the control group and 20% RNA from experimental groups exposed to the 3 concentrations of niclosamide. All experimental samples contributed equally to the URR.

All procedures related to the microarray experiment were based on Agilent’s two-color microarray-based gene expression analysis (Version 6.6). Unless otherwise mentioned below, all reagents were purchased from Agilent Technologies and were used in accordance with the manufacturer’s protocol.

A total of 200 ng RNA was used for each biological replicate of the four unknown groups (control snails or snails exposed to the three different dilutions) to be examined. Spike A buffer mix was added to the unknown RNA sample and Spike B buffer mix was added to 200 ng of the URR. Complementary RNA (cRNA) amplification and labeling reactions with cyanine dye Cy–3 in the unknown sample and cyanine dye Cy–5 in the URR were conducted at 42°C for 2 hours. The labeled cRNA was purified using the RNeasy Mini Kit (Qiagen). The cRNA was quantified to determine the concentration and specific-labeling efficiency using a NanoDrop spectrophotometer.

A total of 300 ng of both the purified Cy–3 and Cy–5 labeled cRNA was added to 5 μl of 10x hybridization buffer and incubated at 60°C for 30 min to fragment RNA. A total of 40 μl of hybridization solution (representing a single replicate for a particular unknown sample) was added to one of the eight gasket chambers. Each of the eight available gasket chambers was in this way filled with a different sample. The gasket surrounding the eight chambers was then covered with a microarray slide such that each of chambers contacted one of the eight different identical arrays printed on that slide. Two slides were used for this study, each with eight identical arrays. The 16 arrays thereby accommodated the 16 different samples that needed to be run (4 unknowns by 4 replicates per unknown). The assembled hybridization chamber was placed in a hybridization oven at 65°C for 17 hours (rotating at 10x*g*).

After hybridization, the slides were washed in Agilent’s wash buffer I for 1 min (room temperature), then in wash buffer II for 1 min (37°C). After stabilizing against ozone deterioration in stabilization buffer, the slides were dried and shipped to the University of California San Francisco’s Viral Diagnostics and Discovery Center, where they were scanned with Agilent’s Microarray G2505C Scanner and extracted and normalized using Agilent’s Feature Extraction software.

### Microarray data analysis

Microarray data were analyzed using GeneSpring GX version 12.6.1 (Agilent). For quality control, the program called “Filter probsets on data” was used. After removing all control probes, experimental probes were retained for further analyses only when they were expressed in at least 75% of all replicates from at least one biological condition (experimental or control). The features that were not positive, not significant, not above background noise, not uniform, not saturated or that were population outliers were not analyzed further. The remaining features were analyzed using volcano plots. Benjamin Hochberg FDR was applied to correct for multiple testing. Corrected *P*-value cut-off and fold change cut-off were set at 0.05 and 2, respectively. In accordance with MIAME guidelines, all relevant data were deposited in the GEO database at NCBI (accession GSE71223).

### Blast search, gene ontogeny (GO) and heatmap analyses

The features that passed the statistical criteria (2 fold change and *P*< 0.05) were selected for subsequent analyses. The full-length transcripts associated with probes (see GEO platform accession GPL20716) that showed differential expression were selected for Blastx search and GO analysis (Blast2Go v.5; www.blast2go.com). The three GO term categories (biological process, molecular function and cellular component), all at the level 2, were generated using the program.

All heatmaps were done in R Studio version 0.98.953 [33–36]. The transcripts that were differentially expressed were categorized according to GO terms (http://geneontology.org/) and UniProtKB (http://www.uniprot.org/) functional descriptions. Many of the proteins shown could be placed in multiple functional categories, but for simplicity we have included each protein in only one category.

## Results

The primary objective of the study was to use microarray analysis to reveal differential gene expression in response to 24-hr exposure to three sublethal concentrations of niclosamide. The highest concentration (0.15 mg/L) resulted in 21% mortality whereas the two lower concentrations (0.10 mg/L and 0.05 mg/L) did not cause any mortality. These results agreed with mortality data reported previously [37,38].

**Fig 1:**
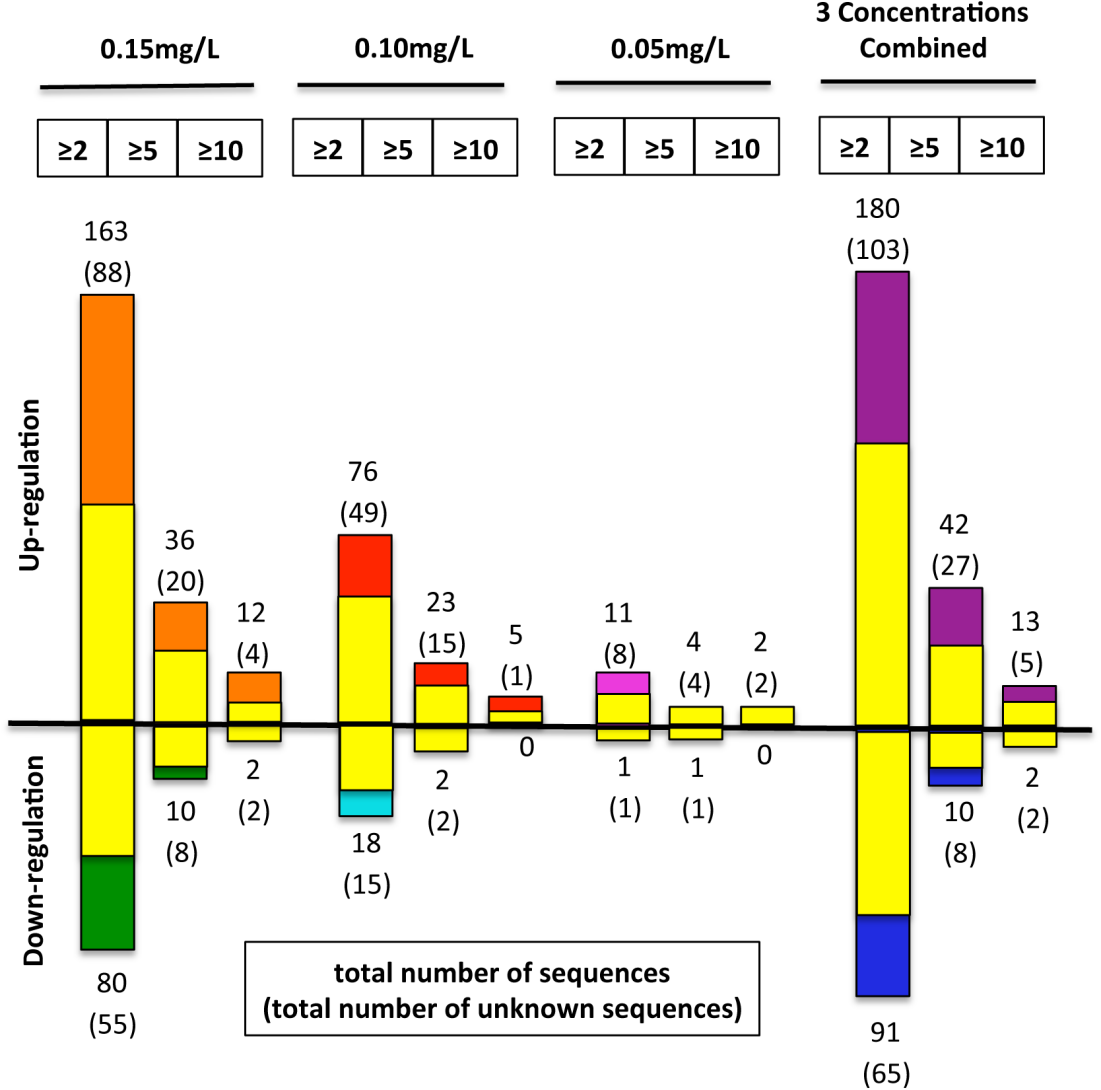
Numbers of sequences that were up- or down-regulated in snails after exposure to the three concentrations of niclosamide. For each concentration, the total numbers of sequence changes in different folds are provided. In addition, the numbers of unknown sequences are also provided (yellow color). Numbers in boxes at the top of the figure refer to fold change for each dose.

**Fig 2:**
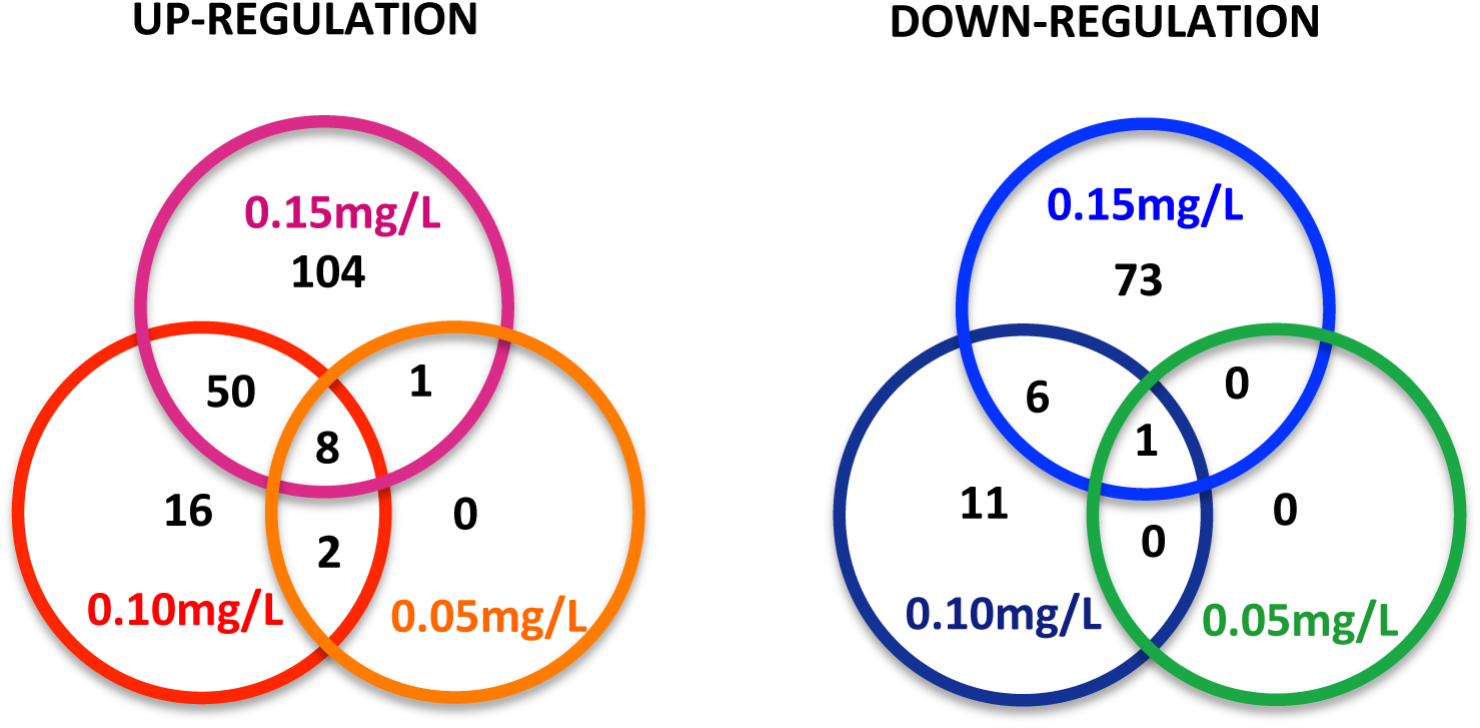
Diagram showing the number of sequences among the three experimental groups that were up- or down-regulated.

Of 30,647 probe features, 24,778 features (81%) were used for analysis after quality control and filtration measures had been applied. The number of genes differentially expressed was positively related to niclosamide concentration: 12 at 0.05 mg/L, 94 at 0.10 mg/L, and 243 at 0.15 mg/L (Fig 1 and S1 and S2 Tables). In combining the three concentrations, 272 genes were differentially expressed, with 181 upregulated and 91 downregulated (all statistically significant). Nine genes were responsive at all three concentrations, eight of which were upregulated and one of which was consistently downregulated (Fig 2). BlastX search revealed that 43% (78 of 181) of upregulated genes and 29% (26 of 91) of downregulated genes have homologs with putative functions in other animals (Fig 1).

**Fig 3:**
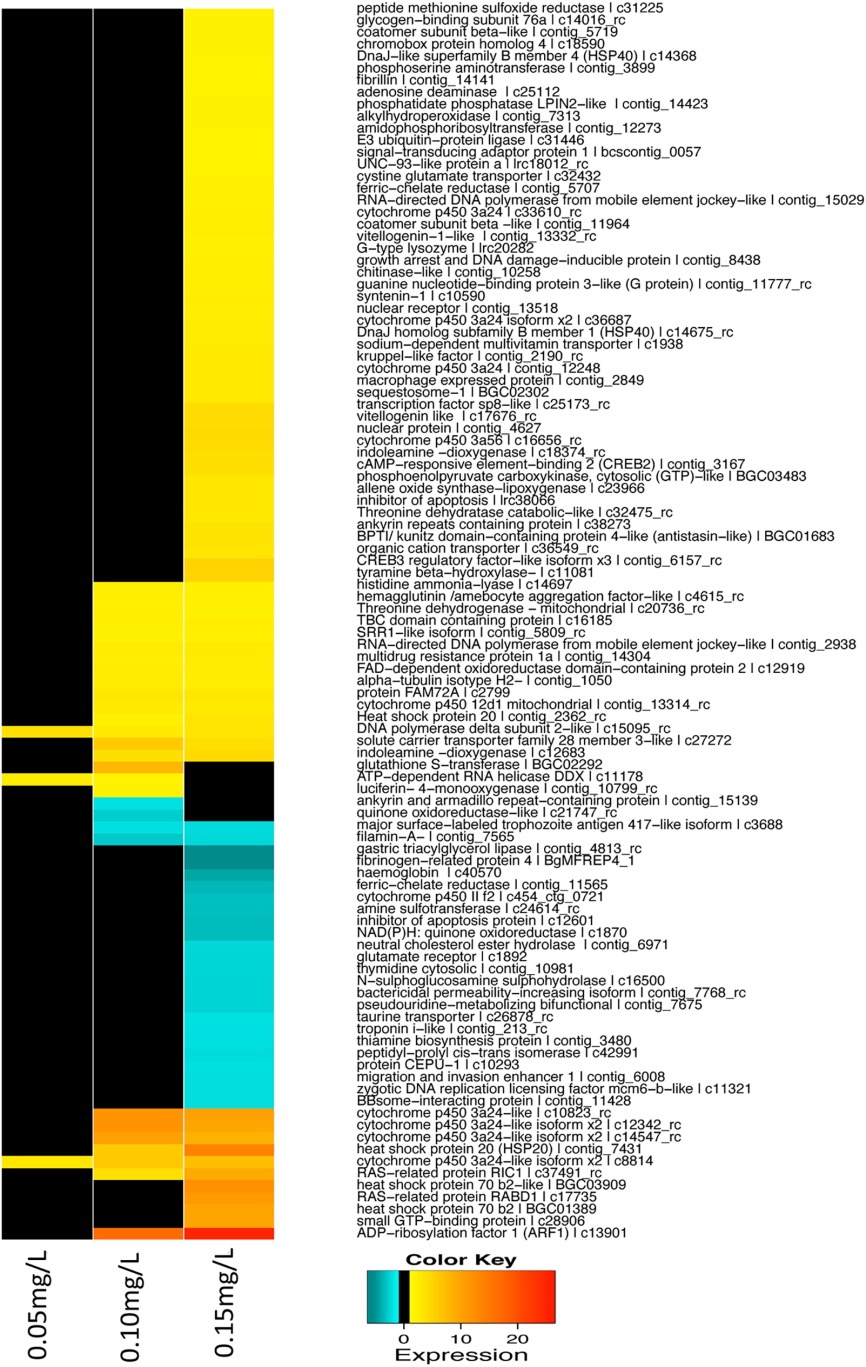
Hierarchical clustering of expression values from annotated genes significantly differentially expressed at all niclosamide concentrations (0.05mg/L, 0.10mg/L, and 0.15mg/L).

Table 1 provides a list of genes with putative GenBank homologs that are either significantly up- or downregulated, including an estimate of fold-change in expression, a significance level, and a sequence description with a Blast score (see also S1 and S2 Tables). Fig 3 provides an overview of known genes differentially expressed in the snails after exposure to the three concentrations. It is noteworthy that out of 78 transcripts with ≥2 fold over-expression, 9 were cytochrome P450s (CYPs) and 6 were heat shock proteins (HSPs). Of the genes for which a reasonable inference could be made with respect to function, several are known to be involved in biotransformation of xenobiotics and stress responses (see discussion below). Only 26 genes with homologs in GenBank were downregulated after exposure to sublethal concentrations of niclosamide.

**Table 1:** List of differentially expressed transcripts that have putative homologs in GenBank.

**UP REGULATION**				
Probe	Fold changes	P value	Sequence description	E value
c13901	27	3.02E-05	ADP ribosylation factor 1 (ARF1)	2.56E-52
contig_7431	14	5.71E-05	heat shock protein 20 (HSP20)	2.21E-24
BGC03909	12	9.59E-05	heat shock protein 70 b2-like	1.52E-84
c10823_rc	12	1.60E-05	cytochrome p450 3a24-like	1.01E-28
c12342_rc	12	5.38E-06	cytochrome p450 3a24-like isoform x2	4.82E-50
c17735	11	2.51E-05	RAS related protein RABD1	1.57E-46
c14547_rc	10	1.17E-04	cytochrome p450 3a24-like isoform x2	3.74E-18
BGC01389	10	1.98E-05	heat shock protein 70 b2	0
c28906	10	1.76E-04	small GTP binding protein	1.24E-09
c37491_rc	9	1.03E-05	RAS related protein RIC1	3.14E-10
BGC02292	8	1.86E-04	glutathione S-transferase	1.99E-41
c8814	7	2.47E-08	cytochrome p450 3a24-like isoform x2	1.58E-49
c27272	6	1.59E-05	solute carrier transporter family 28 member 3-like	1.39E-33
contig_6157_rc	5	1.37E-04	CREB3 regulatory factor-like isoform x3	7.01E-28
c11081	5	4.63E-04	tyramine beta-hydroxylase-	8.38E-96
c12683	5	4.38E-05	indoleamine dioxygenase	1.46E-13
contig_4627	5	3.13E-05	nuclear protein	1.72E-25
c25173_rc	5	2.92E-04	transcription factor sp8-like	2.41E-06
SmM_ND5	4	2.89E-04	NADH dehydrogenase subunit 5	0
c17676_rc	4	8.19E-05	Vitellogenin-like	9.87E-14
c16656_rc	4	2.63E-04	cytochrome p450 3a56	6.50E-13
c18374_rc	4	7.48E-04	indoleamine dioxygenase	6.10E-17
contig_3167	4	7.71E-05	cAMP responsive element binding 2 (CREB2)	6.34E-26
c38273	4	9.22E-04	ankyrin repeats containing protein	2.17E-20
BGC01683	4	2.44E-04	BPTI/ kunitz domain containing protein 4-like (antistasin-like)	7.40E-06
c15095_rc	4	8.89E-07	DNA polymerase delta subunit 2-like	6.94E-42
c36549_rc	4	4.31E-05	organic cation transporter	2.68E-18
contig_2362_rc	4	2.72E-06	heat shock protein 20	3.33E-12
c20736_rc	3	1.20E-04	threonine dehydrogenase—mitochondrial	1.11E-61
c11178	3	1.31E-05	ATP dependent RNA helicase DDX	4.32E-47
c2799	3	7.18E-05	protein FAM72A	8.10E-62
c14697	3		histidine ammonia lyase	1.43E-59
BGC03483	3	3.69E-04	phosphoenolpyruvate carboxykinase, cytosolic (GTP)-like	7.59E-155
c23966	3	9.91E-05	allene oxide synthase lipoxygenase	3.18E-13
lrc38066	3	2.43E-05	inhibitor of apoptosis	6.39E-21
c32475_rc	3	3.88E-04	threonine dehydratase catabolic-like	2.35E-33
contig_2849	3	8.80E-04	macrophage expressed protein	3.05E-85
contig_13314_rc	3	3.41E-05	cytochrome p450 12d1 mitochondrial	2.84E-24
BGC02302	3	1.11E-04	Sequestosome 1	4.02E-15
contig_2938	3	1.03E-04	RNA-directed DNA polymerase from mobile element jockey-like	3.71E-38
c1938	3	6.20E-04	sodium-dependent multivitamin transporter	4.10E-47
contig_2190_rc	3	4.69E-05	kruppel-like factor	9.37E-47
contig_12248	3	4.35E-05	cytochrome p450 3a24	2.12E-52
c36687	3	2.12E-04	cytochrome p450 3a24 isoform x2	2.34E-12
contig_14304	3	2.00E-05	multidrug resistance protein 1a	5.10E-11
c14675_rc	3	6.96E-05	DnaJ homolog subfamily B member 1 (HSP40)	5.54E-55
c12919	3	2.11E-04	FAD-dependent oxidoreductase domain-containing protein 2	3.76E-37
c10590	3	3.53E-05	Syntenin 1	5.78E-23
contig_13518	3	0.001129276	nuclear receptor	8.31E-62
contig_8438	3	1.57E-05	growth arrest and DNA damage inducible protein	2.96E-26
contig_10258	3	5.42E-04	chitinase-like	4.32E-23
contig_11777_rc	3	5.31E-04	guanine nucleotide-binding protein 3-like (G protein)	3.26E-05
contig_1050	3	8.66E-05	alpha-tubulin isotype H2	6.77E-19
c16185	3	3.89E-05	TBC domain containing protein	7.98E-20
c33610_rc	3	3.70E-04	cytochrome p450 3a24	2.54E-09
contig_11964	3	2.57E-05	coatomer subunit beta -like	2.58E-74
c32432	3	0.001228079	cystine glutamate transporter	3.37E-14
contig_5707	3	0.001217638	Ferric chelate reductase	52.20%
contig_15029	3	1.05E-04	RNA-directed DNA polymerase from mobile element jockey-like	3.59E-71
contig_13332_rc	2	1.49E-05	vitellogenin-1-like	1.08E-31
lrc20282	2	5.34E-04	G type lysozyme	5.77E-14
contig_5809_rc	2	7.40E-05	SRR1-like isoform	8.64E-56
c4615_rc	2	8.97E-04	hemagglutinin /amebocyte aggregation factor-like	7.38E-31
c25112	2	5.74E-04	adenosine deaminase	3.06E-26
contig_14423	2	8.34E-04	phosphatidate phosphatase LPIN2-like	7.17E-25
contig_7313	2	6.76E-04	alkylhydroperoxidase	5.45E-89
c31225	2	5.98E-04	peptide methionine sulfoxide reductase	1.73E-07
c14016_rc	2	2.09E-04	glycogen-binding subunit 76a	7.61E-46
contig_5719	2	1.05E-04	coatomer subunit beta-like	5.75E-56
c18590	2	3.59E-04	chromobox protein homolog 4	5.46E-14
c14368	2	0.001067669	DnaJ-like superfamily B member 4 (HSP40)	5.84E-40
contig_3899	2	4.77E-04	phosphoserine aminotransferase	1.39E-121
contig_14141	2	1.96E-04	fibrillin	3.95E-27
contig_12273	2	3.98E-04	amidophosphoribosyltransferase	7.83E-127
c31446	2	4.18E-04	E3 ubiquitin protein ligase	1.32E-05
bcscontig_0057	2	3.77E-05	signal-transducing adaptor protein 1	1.21E-35
lrc18012_rc	2	3.56E-05	UNC 93-like protein a	1.02E-39
contig_10799_rc	2	1.12E-04	luciferin- 4-monooxygenase	1.18E-17
**DOWN REGULATION**				
BgMFREP4_1	6	9.61E-04	fibrinogen-related protein 4	0
contig_4813_rc	6	7.47E-05	gastric triacylglycerol lipase	4.24E-57
c40570	4	0.001065175	haemoglobin	3.57E-10
contig_11565	4	9.50E-04	Ferric chelate reductase	1.52E-11
c1870	4	1.37E-04	NAD(P)H quinone oxidoreductase	2.07E-57
c12601	3	2.17E-04	inhibitor of apoptosis protein	5.68E-12
c24614_rc	3	6.18E-04	amine sulfotransferase	3.85E-53
c454_ctg_0721	3	5.52E-04	cytochrome p450 II f2	1.42E-66
contig_7675	3	6.52E-04	pseudouridine-metabolizing bifunctional	1.55E-54
contig_7768_rc	3	0.001134365	bactericidal permeability-increasing isoform	2.95E-13
c16500	3	2.84E-04	N-sulphoglucosamine sulphohydrolase	4.11E-05
lcl|contig_10981	3	6.07E-04	thymidine cytosolic	8.61E-94
lcl|c1892	3	2.66E-04	glutamate receptor	2.02E-40
contig_6971	3	3.29E-04	neutral cholesterol ester hydrolase	3.08E-51
contig_7565	3	2.53E-04	filamin-A	1.01E-27
c3688	2	7.14E-04	major surface-labeled trophozoite antigen 417-like isoform	
c42991	2	3.61E-04	peptidyl-prolyl cis-trans isomerase	3.50E-09
contig_11428	2	8.11E-04	BBsome-interacting protein 1	2.31E-13
c11321	2	1.63E-04	zygotic DNA replication licensing factor mcm6-b-like	5.36E-43
contig_6008	2	1.08E-04	migration and invasion enhancer 1	3.07E-14
c10293	2	2.14E-04	protein CEPU–1	1.47E-24
contig_3480	2	2.40E-04	thiamine biosynthesis protein	
contig_213_rc	2	5.84E-05	troponin i-like	1.95E-43
c26878_rc	2	4.68E-04	taurine transporter	
c21747_rc	3	2.15E-05	quinone oxidoreductase-like	2.51E-17
contig_15139	2	1.22E-04	ankyrin and armadillo repeat-containing protein	3.57E-37

Note: Genes described in the table were found to be expressed differentially in at least one concentration. If two or more concentrations resulted in differential expression, the higher or highest fold change is given in the table.

With respect to unknown genes lacking similarities to known sequences, 103 were upregulated and 66 downregulated (Fig 1). Among these sequences, several showed very high levels of differential expression. For example, c17544_rc, c7670, and c454_ctg–0279 had up-regulated expression levels of 14-, 13- and 9-fold, respectively (S1 and S2 Tables). Just as with array features with GenBank homologs, for unknown transcripts, generally the fold-changes for downregulated genes were lower than for upregulated genes.

**Fig 4:**
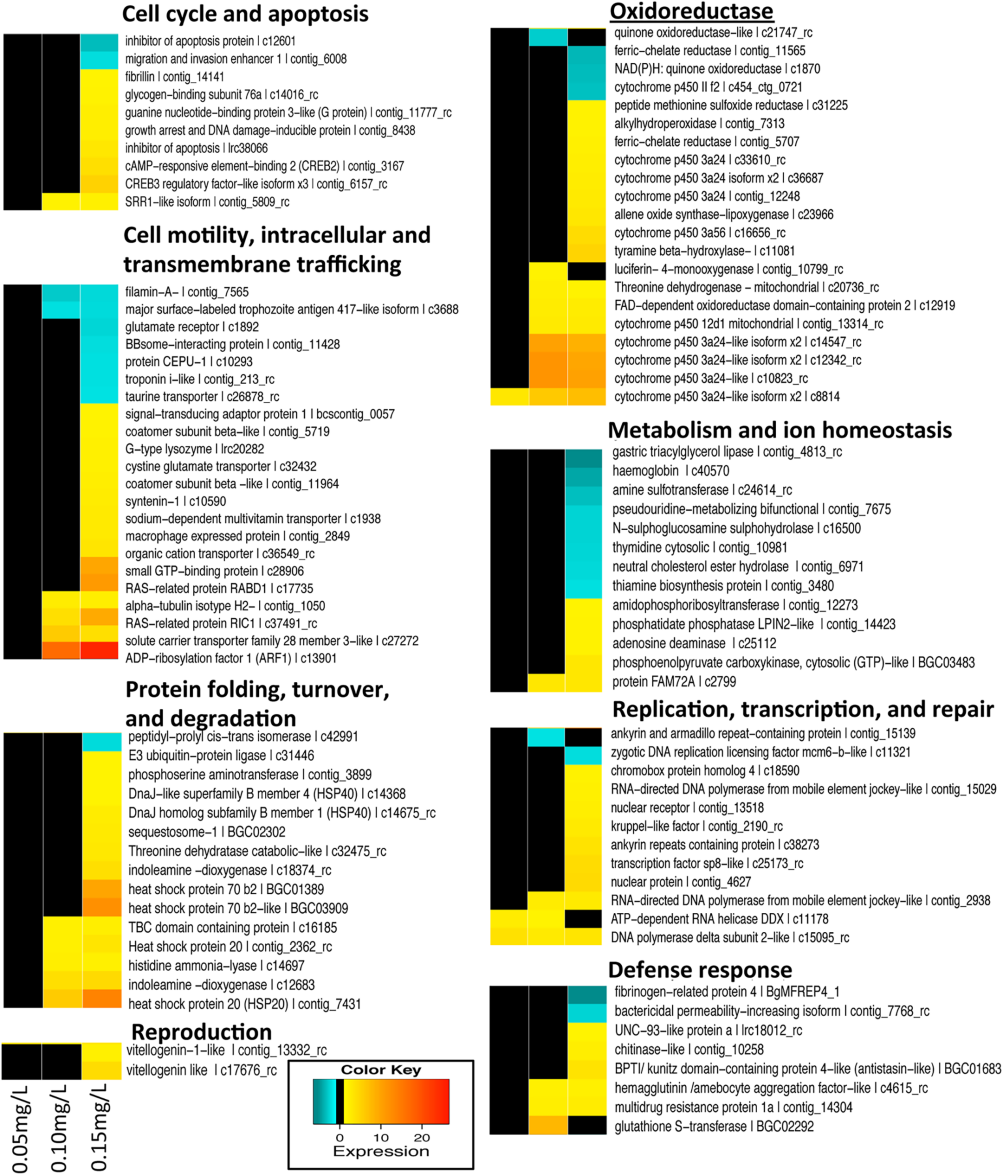
Hierarchical clustering of expression values from annotated genes significantly differentially expressed at all niclosamide concentrations (0.05mg/L, 0.10mg/L, and 0.15mg/L). Transcript descriptions have been reorganized by general functional categories.

When considered with respect to the function (Fig 4), the most responsive genes were in two categories: 1) oxidoreduction, and 2) cell motility, intracellular and transmembrane trafficking. The GO analysis for the three general categories (biological process, molecular function and cellular component), is presented in S3 and S4 Tables.

## Discussion

This study interrogated the most comprehensive *B*. *glabrata* microarray currently available, one that includes enough features to provide a representative overview of the transcripts this snail is capable of producing, including those in response to molluscicides. As such, the array (and the results derived from its use) will provide an appropriate tool to help validate, and provide perspective for next-gen sequencing studies of the responses of *B*. *glabrata* and other schistosome-transmitting snails to molluscicides. Furthermore, unlike next-gen sequencing approaches, microarrays do not require application of assembly software that can introduce errors and produce artifactual chimeric sequences. As the use of molluscicides will increase in the near future, having as many sources of information as possible about their impact on snail biology will be beneficial.

Lipophilic xenobiotics like niclosamide that are readily absorbed into the body are normally eliminated in animals by biotransformation, a process that increases hydrophilicity [39]. Biotransformation includes chemical modification of the xenobiotic by oxidation; reduction or hydrolysis reactions (phase I reactions); conjugation of the phase I metabolite (phase II reactions); and transport of the phase II product (phase III reactions) across epithelial surfaces. When high levels of toxicant accumulate internally, as would be expected in snails continually immersed in niclosamide solution, toxicity can result from interaction of the toxicant with a critical molecule in a target cell. The toxicant or its metabolite may be a strong electrophile, nucleophile, free radical, or redox-active reactant, and may damage the target molecule or interfere with its normal function [40]. In addition to undergoing biotransformation by constitutively-expressed proteins and having immediate toxic effects, a xenobiotic may also alter gene expression. Affected genes may be involved in biotransformation, physiological adaptation to stress, or repair of damage at the molecular, cellular or tissue level. These induced processes are usually protective, e.g., by enhancing detoxification and elimination of the toxicant, allowing compensatory physiological processes to maintain homeostasis, and effecting repair. However, they can also enhance toxicity, e.g., by bioactivation of the xenobiotic to a toxic metabolite, maladaptive physiological responses, or dysrepair, e.g., fibrosis. Consequently, a systems approach provided by the interrogation of microarrays has the potential to provide new insights into the general nature of the complex transcriptomic response to xenobiotics.

### Phase I and phase II biotransformation

Based on studies with better-characterized mammalian models, cytochrome P450 (CYP) enzymes are involved in the most important phase I reactions [35]. CYPs constitute a superfamily of structurally diverse and functionally versatile enzymes with more than 15,000 known genes distributed across all biological kingdoms [41]. CYPs generally catalyze a monooxygenation reaction, in which an atom of oxygen is added to the xenobiotic [39]. In insects, the association of CYP expression with insecticide or drug resistance is well established [42–44]. For example, high expression of a single CYP allele (CYP6G1) confers resistance to DDT in *Drosophila melanogaster* [45] and to pyrethroids in *Anopheles funestus* [46]. Upregulation of CYP expression can be induced by many different xenobiotics, which generally act by combining with an intracellular receptor to form a transcription factor that then binds to xenobiotic response elements of target genes, activating their transcription [39]. Interestingly, the induced CYPs may not be involved in the metabolism of the xenobiotic that causes their upregulation.

In snails CYPs are expected to be diverse, and the array used for this study contains about 100 distinct features with similarity to CYP genes, but thus far, we know little of the overall diversity of CYP genes in *B*. *glabrata*, and their full range of functions. In this study, 9 CYPs were upregulated, and one was downregulated in niclosamide-exposed snails (Table 1 and Fig 4). Out of 16 transcripts with ≥5 fold upregulation, 4 were CYPs, indicating they are an important component of the snail transcriptomic response following exposure to niclosamide. Only one full-length cDNA of a CYP (AY922309) has been previously reported from *B*. *glabrata*, and exposure of snails to *S*. *mansoni* resulted in downregulation of its expression [47]. Since sequences of CYPs are highly diverse [41] and only partial sequences of the 10 differentially expressed CYPs observed in the study are available, a phylogenetic analysis is unlikely to reveal reliable relationships among the snail CYPs discussed. However, the complex expression pattern of the 10 CYPs presented in this study as well as altered expression of one CYP (AY922309) in response to *S*. *mansoni* suggest CYPs have diverse functions in *B*. *glabrata*.

Provision of additional functional information regarding the specific CYPs induced by niclosamide or other molluscicides is important, because it will provide further needed details regarding the detoxification process in snails, and could potentially lead to an ability to select for compounds with higher specific activity for snails relative to other aquatic organisms. Also, prior to widespread application of molluscicides, it would be of interest to identify the array of CYPs present, to facilitate subsequent monitoring for the possible emergence of molluscicide-resistant snails that might express mutated CYPs as has been documented in insects. Thus far there has been no clear indication for the emergence in snails of resistance to niclosamide [48], although Sullivan et al. (1984) reported an approximately two-fold higher tolerance to niclosamide in a laboratory strain of *B*. *glabrata* after 5 generations of selection [49].

Relative to phase II (conjugation) proteins, our study revealed one glutatione S-transferase (GST) that was 8-fold overexpressed following niclosamide exposure. Like CYPs, GSTs are also markers of metabolism of xenobiotics and indicators of the presence of contaminants [50]. Based on Illumina RNA-seq analysis, Zhao et al. (2015) described the responses of the amphibious snail, *Oncomelania hupensis*, after challenge with two different niclosamide-based molluscicides. They showed that two CYP genes and one GST gene were upregulated following molluscicide exposure [51]. This observation coupled with our results suggests that both enzyme families are a common component of the snail transcriptomic response to molluscicides. In contrast, GSTs but not CYPs were shown to be highly expressed in a microarray study of the gills of the mussel *Mytilus galloprovincialis* following exposure to salts of heavy metals [52]. This suggests that molluscs can respond differently depending on the nature of the toxicant.

### Phase III biotransformation

Drug transporters play a vital role in translocation of compounds such as nutrients, wastes, toxins and xenobiotics into or out of cells [53–55]. The transporters work in conjunction with drug metabolizing enzymes such as CYPs and phase II enzymes for drug elimination. They can be classified as influx and efflux transporters, which are located either at the basolateral or apical membranes.

Efflux transporters are ATP-binding cassette (ABC) transporters that belong to a superfamily including multidrug resistance proteins (MRP). These efflux pumps determine bioavailability and concentrations of many drugs. In our study, a highly expressed MRP was found (contig_14304). MRPs preferentially transport anionic compounds and compounds detoxified by cellular enzymes such as GST.

Influx transporters are members of the solute linked carriers (SLC) superfamily responsible for transporting organic anions, organic cations or oligopeptides [56]. In this study, two transcripts that encode solute carrier transporter family 28 (c27272) and organic cation transporter (c36549_rc) have also been shown to be 6- and 4-fold upregulated, respectively, following niclosamide exposure (Table 1).

In addition, we have noted that a sodium-dependent multivitamin transporter (c1938) and a cystine-glutamate transporter (c32432), two additional influx transporters, were also highly expressed. Sodium-dependent multivitamin transporter is an important transmembrane protein responsible for translocation of vitamins and other essential cofactors such as biotin. Vitamins are required for detoxification metabolism and vitamin E (α-tocopherol) is involved in repair of peroxidized lipids [40]. Cystine-glutamate transporter mediates cystine entry in exchange for intracellular glutamate in mammalian cells. Cystine is converted to cysteine, which is required for synthesis of glutathione, an antioxidant that prevents damage due to reactive electrophiles.

Transporters are well-known for their roles in drug efficacy and resistance [57]. ABC transporters are upregulated in schistosomes in response to praziquantel [58,59]. Knockdown of ABC transporters enhances susceptibility of adult and juvenile schistosomes to praziquantel [60]. In addition, ABC transporters play a critical role in diverse physiological functions in schistosomes including immune responses and reproduction [55]. The role of transporters in the response of snails to molluscicides awaits further study.

In summary, genes encoding key molecules involved in all three phases of biotransformation in *B*. *glabrata* were responsive to molluscicide treatment and provide a basis for beginning to understand the molecular basis of detoxification in freshwater snails.

### Stress response

The stress response is characterized by the production of stress proteins which tend to be relatively well-conserved across both prokaryotes and eukaryotes. Often it is the presence of denatured proteins that triggers a stress response, including production of heat shock proteins (HSP). HSPs, mainly acting as molecular chaperones, are involved in protein folding, assembly, degradation, and intracellular localization. Under normal conditions, HSPs are constitutively expressed. Heightened expression is triggered by various physiological perturbations or stressors (e.g. elevated temperature, hypoxia, ischemia, heavy metals, radiation, calcium increase, glucose deprivation, pollutants, drugs, cancer, and microbial infection) [61]. Functions within the HSP superfamily are highly diverse. We found six HSPs belonging to three families (HSP20, HSP40 and HSP70) to be upregulated upon exposure to niclosamide (Table 1 and Fig 4). High molecular weight HSPs such as HSP90s were not differentially expressed.

HSP20s protect other proteins against heat-induced aggregation or denaturation. HSP20 was identified as a biomarker for environmental stress in the disk abalone, *Haliotis discus discus*, and its expression could be induced by extreme temperatures, salinities, heavy metals and microbial infection [62]. HSP90, HSP70, HSP24.1 and sequestosome–1 were also highly expressed in the marine bivalve *Mytilus galloprovincialis* exposed to toxic metals [54]. The latter gene encodes a protein involved in ubiquitin binding and is therefore related to proteasome degradation. We also found this gene to be upregulated in *B*. *glabrata* exposed to niclosamide. HSP40s, also referred to as DnaJ/Hsp40, stimulate the ATPase activity of chaperone proteins, HSP70s, by stabilizing their interactions with protein substrates [63].

In addition to stress responses, HSPs are also involved in detoxification, immune responses, pathogenesis and cancer development [64,65]. In *Biomphalaria*, an increased expression of HSPs has been linked to susceptibility of snails to schistosome parasites [66–68]. The relationships between the HSPs discussed in these studies and the 6 HSPs presented in this study are unclear because complete cDNA of the HSPs we studied are lacking. Eventual comparisons of full-length HSP cDNAs will help resolve this matter.

### Intracellular and transmembrane trafficking

The most highly expressed gene we noted following niclosamide exposure was the gene encoding ADP-ribosylation factor 1 (ARF1). ARF1 is a member of the family of GTPases, and a key regulator of intracellular vesicle trafficking at the Golgi apparatus and endosomes. At the Golgi complex, ARF1 facilitates membrane recruitment of many cytoplasmic coat proteins to allow sorting of membrane proteins for transport. It also stimulates the activity of enzymes that modulate the lipid composition of the Golgi, and that assemble cytoskeletal scaffolds on the Golgi [69–71]. A recent study demonstrated that ARF1 upregulation, apparently coupled to ARF4 downregulation, enables Golgi secretory pathway activity to continue even in the presence of inhibitors [72]. This effect may involve an ARF-CREB signaling pathway, which may be affected by a CREB3 regulatory factor-like isoform, which acts as a negative regulator of the ER unfolded protein stress response. The upregulation of CREB3 regulatory factor-like isoform we observed may then interfere with the production of some ARF molecules like ARF4, which could be compensated for by the production of others, like ARF1 [72].

In eukaryotic cells membrane compartments are connected through cargo-selective vesicle trafficking, thereby mediating the exchange of components between different organelles. This exchange is essential to maintain structural integrity and specific composition [73]. A fundamental regulatory step in vesicle formation is the activation of small ARF GTPases by exchanging their bound GDP for GTP, which is a prerequisite for ARF-mediated effector recruitment [74]. In our study, in addition to ARF1, four additional GTPases (c17735, c28906, c37491_rc, and BGC03483), all with a conserved ABC-ATPase domain, were over-expressed in niclosamide-treated snails.

Other recent studies also suggest that ARF1 plays an important role in modulating the morphology and function of mitochondria [75–76]. Mitochondria and ER are found in close proximity to each other and it is thought that they maintain contact sites to facilitate exchange of molecules. ARF1 is well known for its essential role in the generation of coatomer protein I-coated vesicles in the Golgi complex, which are important for maintenance of mitochondrial function, possibly at ER-mitochondrial contact sites. Indeed, our study has revealed that two coatomer genes were over-expressed following niclosamide treatment (Table 1). Thus, the upregulation of ARF1, coatomer, and GTPase genes may be an important compensatory response to toxic damage, allowing maintenance of Golgi and mitochondrial function.

In contrast to a potential role in preserving organelle function, GTPases can be involved in a process involving mitochondrial fragmentation. Mitochondria form a highly dynamic network throughout the cell, which is maintained through constant fission and fusion of mitochondrial tubules. These fission and fusion events are regulated by two types of GTPases in mammalian cells [77]. Park et al. (2011) showed that niclosamide was a potent inducer of mitochondrial fragmentation in human HeLa cells, resulting in the disruption of mitochondrial membrane potential, reduction of adenosine triphosphate (ATP) levels, and cell death, and that this fragmentation was mediated by the GTPase Drp1 [78]. Whether the 4 GTPases upregulated in our study have a protective or cytotoxic role requires further study.

### Genes downregulated following niclosamide exposure

One of the most conspicuously downregulated genes with known homologs in GenBank was *FREP4*. FREP4 is involved in immune responses to trematodes [79] as well as in ontogenesis [80]. FREPs belong to a gene family with many members, and the functions of its members are expected to be diverse [81–83]. Other studies have also noted underexpression of immune genes in animals subjected to toxin or xenobiotic pressure. Varotto et al. (2013) found that several transcripts associated with immunity including fibrinogen-containing protein gene 7 were under expressed in *M*. *galloprovincialis* exposed to toxic metals [52]. In strains of the mosquito *Aedes aegypti* resistant to *Bacillus thuringiensis israelensis* toxins, genes involved in immune responses were generally under-transcribed [84], and exposure of sphingid caterpillars to toxic plant compounds was shown to weaken their melanization immune responses [85].

In planorbid snails like *B*. *glabrata*, hemoglobin plays the primary role in transporting oxygen and is an abundant hemolymph protein, so it is noteworthy that a four-fold reduction in hemoglobin gene expression was observed. It is clear that snails like lymnaeids that rely on hemocyanin as a respiratory pigment are also affected by niclosamide [7], so it seems unlikely that depressed hemoglobin synthesis per se could be the only mechanism of niclosamide toxicity in *B*. *glabrata*. As noted above, early investigations demonstrated that niclosamide affects oxygen uptake in *B*. *glabrata*, with higher concentrations resulting in the inhibition of oxygen intake [13]. Downregulation of hemoglobin gene expression may be one reason for decreased oxygen uptake noted in such studies, which in turn could contribute to toxicity.

### Direct effects of niclosamide versus indirect toxic effects

Hypothetically, at the organismal level changes in gene expression following exposure to a toxicant can result from direct as well as indirect mechanisms. Specifically, changes can occur in target cells that directly interact with a toxicant, or can occur in cells that instead are responding to non-toxicant molecules (e.g., from necrotic, inflammatory, or neuroendocrine cells) or to toxicant-altered physiological parameters (e.g., hemolymph pH, osmolarity, or O_2_ concentration). If concentration increases to a level causing wholesale death of target cells, then we would expect indirect mechanisms to become more pronounced. With increasing concentrations of niclosamide, we observed an increase in the number of both upregulated and downregulated genes, with a predominance of upregulated genes. Some of the changes noted at higher concentrations may indeed be the result of indirect toxic effects However, the known functions of responsive genes and the overall patterns observed are clearly suggestive of the involvement of protective biological processes that are affected by niclosamide. The responsive genes comprise a list of candidate targets for niclosamide and offers leads for future development of novel molluscicides.

### Conclusion

Our data suggest that niclosamide alters expression of several genes involved in biotransformation, stress responses, intracellular organelle trafficking and oxygen transport. The aggregate toxicological significance of these altered expression patterns is not clear, but it is reasonable to speculate that some of these transcriptomic responses are adaptive, allowing enhanced elimination of niclosamide and perhaps maintenance of protein and organelle function and repair, whereas others may lead to enhanced toxicity, e.g., by suppressing immune function, lowering oxygen carrying capacity of hemolymph and contributing to mitochondrial damage. Future studies of the effects of niclosamide on snails need to address which of the transcriptomic responses are key to understanding its mechanism of toxicity. Such responses can then serve as useful biomarkers for testing new candidate molluscicides. Also, it will be important to distinguish between direct effects of niclosamide on target cells and indirect effects, and to assess the role of these effects in niclosamide toxicity at low and high concentrations. Finally, genes expressed in snails during recovery from exposure may provide insights on protective detoxification and repair mechanisms that can serve as future targets.

A long-term goal of molluscicide research is to develop chemicals that have more specific effects on targeted snail species, without widespread toxicity to nontarget organisms such as fish, which are killed by niclosamide. Previous studies have shown that mammalian and tapeworm mitochondria differ markedly in their responses to niclosamide, thereby accounting for the selective toxicity of this drug against tapeworms and its low toxicity in mammals [86]. This observation raises the possibility that mitochondrial or other cellular functions in snails might also be different and potentially more vulnerable to more specific molluscicides that leave aquatic vertebrates unaffected. Deciphering such differences with the use of molecular tools should help in the rational design of a next generation of highly specific molluscicides.

## Supporting Information

S1 Table The details of BlastX results showing fold changes and putative homologs of 181 upregulated sequences in the snails after exposure to three sublethal concentrations of niclosamide. (XLS)

S2 Table The details of BlastX showing fold change and putative homologous sequences of 91 downregulated sequences in snails after exposure to the three concentrations of niclosamide. (XLS)

S3 Table GO terms of all upregulated sequences (at level 2): Biological process, molecular function and cellular component. (XLS)

S4 Table GO terms of all downregulated sequences (at level 2): Biological process, molecular function and cellular component. (XLS)
